# Punicic acid alleviates methylglyoxal-induced oocyte dysfunction during in vitro maturation in mouse species

**DOI:** 10.1371/journal.pone.0314602

**Published:** 2025-03-25

**Authors:** Shahrzad Ronasi, Amir Hossein Mahdavi, Shiva Rouhollahi Varnosfaderani, Rasoul Kowsar, Farnoosh Jafarpour, Mohammad Hossein Nasr-Esfahani

**Affiliations:** 1 Department of Animal Science, College of Agriculture, Isfahan University of Technology, Isfahan, Iran; 2 Department of Animal Biotechnology, Reproductive Biomedicine Research Center, Royan Institute for Biotechnology, ACECR, Isfahan, Iran; School of Pharmacy, Ardabil University of Medical Sciences, IRAN, ISLAMIC REPUBLIC OF

## Abstract

Dicarbonyl stress, characterized by the abnormal accumulation of reactive dicarbonyl metabolites and advanced glycation end-products (AGEs), is implicated in various pathological conditions, including obesity, diabetes, and reproductive disorders. Methylglyoxal (MGO), a highly reactive dicarbonyl metabolite, has been shown to compromise oocyte quality and developmental competence. In this study, we investigated the protective role of punicic acid (PA), a potent antioxidant found in pomegranate seed oil, against MGO-induced oocyte dysfunction. Our findings revealed that 75 µM MGO exposure during *in vitro* oocyte maturation significantly reduced the maturation rate and impaired subsequent embryonic development, characterized by decreased pronucleus formation and blastocyst rates. Interestingly, PA supplementation partially ameliorated these adverse effects of MGO, highlighting its potential as a protective agent against dicarbonyl-induced oocyte dysfunction. Co-treatment with PA restored the imbalanced redox state induced by MGO, leading to reduction in ROS levels and an increase in GSH levels in matured oocytes. Additionally, co-supplementation with PA preserved mitochondrial distribution in oocytes challenged with MGO, further contributing to improved oocyte quality. At the molecular level, PA co-treatment modulated the expression of genes involved in dicarbonyl stress and oxidative responses, including *Glo1*, *Rage*, *Nrf2*, and *Nf-*κ*B*, potentially regulating the detoxification of MGO and mitigating its harmful effects. Lastly, PA supplementation improved cell lineage allocation in blastocysts developed from MGO-challenged oocytes, emphasizing its role in enhancing the quality of preimplantation embryos. In conclusion, our study provides novel insights into the protective effects of punicic acid as an antioxidant against MGO-induced oocyte dysfunction, suggesting its potential as a dietary intervention to enhance reproductive health, particularly in individuals facing dicarbonyl stress-associated conditions such as obesity and diabetes.

## Introduction

Dicarbonyl metabolites are formed via nonenzymatic glycoxidation reactions and induced by the nucleophilic addition of free amino groups from proteins, lipids, or nucleic acids to carbonyl groups of monosaccharides. The Maillard reaction, commonly referred to as nonenzymatic browning, results in the formation of reversible Schiff bases that spontaneously rearrange into reversible Amadori products. Subsequently, these Amadori products undergo an irreversible reaction, leading to the formation of reactive dicarbonyls compounds [1]. Methylglyoxal (MGO) is the most reactive dicarbonyl metabolite in physiological systems [2]. Dicarbonyl stress, characterized by the abnormal accumulation of dicarbonyl reactive metabolites leads to increased protein and DNA modification, contributing to cell and tissue dysfunction in disease and aging [3].

MGO undergoes a series of oxidation and dehydration reactions, eventually converting into stable products known as advanced glycation end-products (AGEs) [4]. Previous studies have shown that the cumulative presence of both reactive dicarbonyl compounds and AGEs is associated with various pathological conditions, including obesity, polycystic ovarian syndrome (PCOS), diabetes and aging [5,6].

AGEs originate from two main sources: endogenous and exogenous. The production of endogenous AGEs significantly accelerates under hyperglycemia and hyperlipidemia conditions [7]. Despite the general conception, exogenous AGEs contribute more to the overall compared to endogenous AGEs. Major sources of exogenous AGEs include foods cooked at high temperatures, such as baking, frying, and stir-frying. Consequently, the modern dietary habits lead to an increased accumulation of AGEs in the body, closely linked to health implications [8].

The adverse effects of AGEs accumulation in the intracellular space are mediated by a cell surface receptor known as the receptor for AGE (RAGE) [9,10]. Expression of *Rage* mRNA is detected in various tissues of the female reproductive system, including the ovary, fallopian tube, endometrium, placenta, etc [11,12]. Under physiological conditions, RAGE expression levels are low in most organs. However, certain pathophysiological conditions, such as obesity, PCOS, and diabetes, can lead to an upregulation of RAGE expression [10,11,13].

AGE-RAGE/MGO-RAGE interactions disrupt the reduction-oxidation balance in biological systems, triggering oxidative responses [13,14]. Oxidative stress, a consequence of dicarbonyl stress from both endogenous (obesity, diabetes, PCOS, and aging) and exogenous sources (Food and beverages) results in elevated level of ROS, mitochondrial malfunction, and DNA damage.

Another consequence of dicarbonyl stress is the depletion of the intracellular levels of GSH.

In the glyoxalase system, MGO reacts with GSHto forme a compound known as S-d lactoylglutathione, which is Subsequently processed by glyoxalase 1 (GLO1)., Following this, glyoxalase 2 (GLO2) catalyzes the release of D-lactate, thereby facilitating the recycling of trapped GSH [15].

It has also been shown that AGE-RAGE/MGO-RAGE interactions activate a series of inflammatory cascades, including mitogen-activated protein kinase (MAPK), extracellular signal-regulated kinase1/2 (ERK1/2), protein kinase C (PKC) and nuclear factor kappa B (NF-κB) [6]. In addition, MGO/AGE can interact with Toll-like receptor 4 (TLR4) and trigger inflammatory signaling pathways [16].

Recently, nutritional interventions have emerged a promising approach for the treatment of metabolic disorders such as obesity, PCOS, and diabetes [17]. Polyunsaturated fatty acids (PUFAs) have been identified as having significant health benefits, providing new insights into their role in metabolic health. PUFAs are categorized into conjugated and non-conjugated forms. Previous studies have shown that conjugated linolenic acid has a higher antioxidant capacity compared to the non-conjugated form due to its specific rearrangement of the molecular structure that makes it more stable and rigid and allows it to donate electrons to neutralize free radicals efficiently [18]. Studies indicate that conjugated linolenic acid, particularly its isomer Punicic acid (PA) found in pomegranate seed oil, possesses antioxidant, anti-inflammatory, anti-obesity, and anti-diabetic properties. The antioxidant properties of PA were confirmed in several experimental studies. PA attenuates oxidative stress via several potential mechanisms, including inhibiting ROS generation, increasing glutathione peroxidase (GPX) levels, reducing inflammatory cytokines production, and malondialdehyde (MDA) levels [19–24].

Research in the field of MGO/AGE has increasingly focused on the effects of dicarbonyl stress on reproductive aspects. This study aims to investigate the potential of PA to mitigate the detrimental effects of MGO during *in vitro* oocyte maturation. To elucidate the underlying mechanisms, we assessed preimplantation embryonic development, redox state, mitochondrial function, and the relative mRNA expression of relevant genes, including *Glo1*, *Rage*, *Nrf2*, and *NF-κβ* in mature cumulus-oocyte complexes (COCs). Our findings demonstrate that MGO compromises the quality of matured oocytes regarding redox state and mitochondrial distribution, resulting in lower preimplantation embryonic development. Notably, Punicic acid might mitigate the adverse effects of MGO, offering a novel approach to improving the quality of MGO-challenged oocytes. Given the protective role of cumulus cells in safeguarding oocytes against various stressors, particularly oxidative stress, this study not only evaluates the effects of MGO on cumulus oocyte complexes (COCs) but also examines its effect on denuded oocytes (DOs) These findings may have implications for understanding the malfunction of the female reproductive system in individuals challenged with obesity and other conditions associated with elevated AGEs.

## Materials and methods

### Ethics

All methods employed in this study were following guidelines and regulations approved by the Institutional Review Board and Institutional Ethical Committee of the Royan Institute (IR.ACECR.AEC.1401.065).

### Animals

Inbred NMRI mice (Royan Research Institute, Iran) at 6–8 weeks of age were utilized for obtaining oocytes at the germinal vesicle (GV) stage, employed for COCs or DOs. Oocytes were obtained from superovulated female mice by intraperitoneal injection of 10 IU of pregnant mare serum gonadotropin (PMSG) (GONASER, Spain). The mice were housed in a temperature-controlled environment under a 12 h light/dark cycle (6.00–18.00) and free access to feed and water.

### Experimental design

This study was conducted through a series of four distinct experiments, which are outlined as follows:

#### Experiment 1:

**Fig 1:**
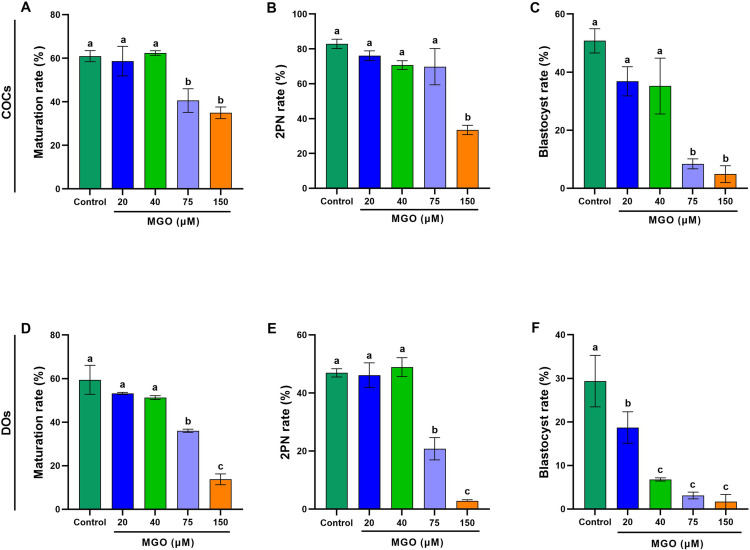
Effect of MGO on *in vitro* maturation and developmental competence of mouse COCs and DOs. Oocytes were collected from PMSG-stimulated NMRI mice, cultured for 18 h in IVM medium containing different concentrations of MGO, fertilized *in vitro*, and transferred to *in vitro* culture medium. A and D) maturation rates, B and E) pronucleus formation rates, and C and F) blastocyst rates in COCs and DOs, respectively. At least 40 COCs and 30 DOs were cultured in each replicate of each treatment group. At least three replicates were included in each treatment group. Data are presented as means ±  standard error mean (SEM). Different letters (a, b and c) indicate significant differences between the treatment groups at *P* < 0.05. Statistical differences between groups were assessed using one-way ANOVA with LSD post-hoc test.

Given the substantial variations in MGO concentrations reported in previous studies, we designed this experiment to identify a threshold concentration of MGO for supplementation in the *in vitro* maturation (IVM) medium that effectively impairs the developmental competence of treated COCs and DOs in terms of maturation rate, 2 pronucleus formation (2PN) rate and blastocyst rate (Fig 1).

#### Experiment 2:

**Fig 2:**
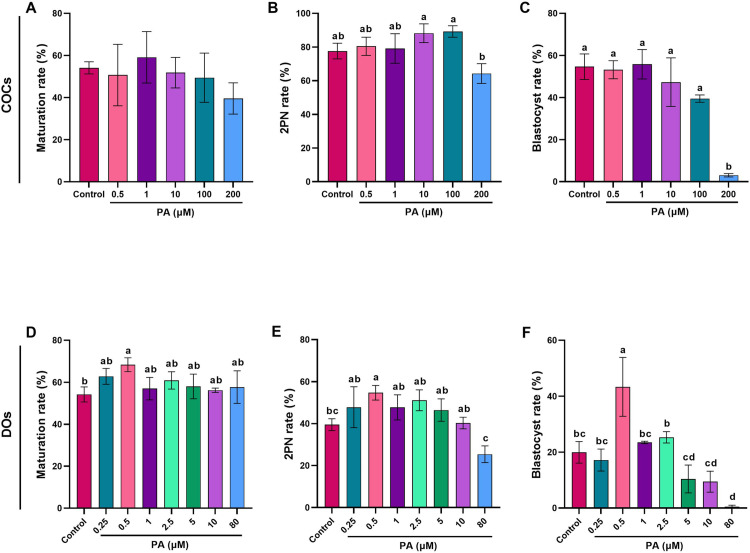
Effect of PA on *in vitro* maturation and developmental competence of mouse COCs and DOs. Oocytes were collected from PMSG-stimulated NMRI mice, cultured for 18 h in IVM medium containing different concentrations of PA, fertilized *in vitro*, and transferred to *in vitro* culture medium. A and D) maturation rates, B and E) pronucleus formation rates, and C and F) blastocyst rates in COCs and DOs, respectively. At least 40 COCs and 30 DOs were cultured in each replicate of each treatment group. At least three replicates were included in each treatment group. Data are presented as means ±  SEM. Different letters (a, b, c and d) indicate significant differences between the treatment groups at *P* < 0.05. Statistical differences between groups were assessed using one-way ANOVA with LSD post-hoc test.

**Fig 3:**
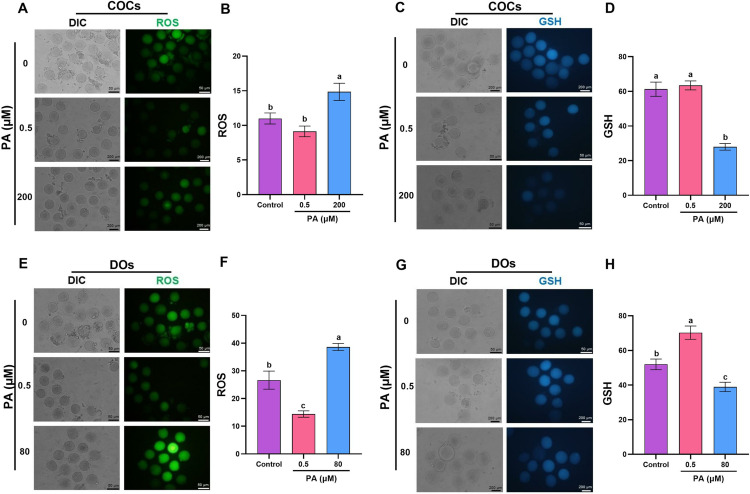
The effect of detrimental and optimal concentrations of PA on ROS and GSH levels of matured COCs and DOs. COCs and DOs were recovered from ovaries of PMSG-stimulated NMRI mice were cultured in IVM medium with indicated concentrations of PA for 18 h, and subsequently were denuded (if needed) for assessment of ROS levels in both COCs (A and B) and DOs (E and F) and also GSH levels in both COCs (C and D) and DOs (G and H). The representative images showed the ROS and GSH levels using a fluorescence microscope. Image J was used to measure the fluorescence intensity of ROS and GSH levels. At least three replicates were included for each assessment and at least 10 oocytes were assessed in each replicate. Data are presented as means ±  SEM. Different letters (a, b and c) indicate significant differences between the treatment groups at *P* < 0.05. Statistical differences between groups were assessed using one-way ANOVA with LSD post-hoc test.

Considering that existing research on PA has primarily focused on various cell lines, and no data is available (up to our knowledge) regarding its optimal concentration within the reproductive system, we designed this experiment to determine the optimum concentration of PA in the IVM medium for COCs and DOs. The evaluation criteria mirrored those of Experiment 1, focusing on maturation rate, 2PN rate, and blastocyst rate. To further investigate the effects of PA, we assessed redox status (ROS and GSH levels) in COCs and DOs treated with both the lowest and highest concentrations of punicic acid (Figs 2, 3).

#### Experiment 3:

We designed this experiment to investigate whether PA (0.5 µM) can alleviate the adverse effects of MGO (75 µ M) during IVM. To this aim, we assessed the redox status and mitochondrial function in matured COCs.

**Fig 4:**
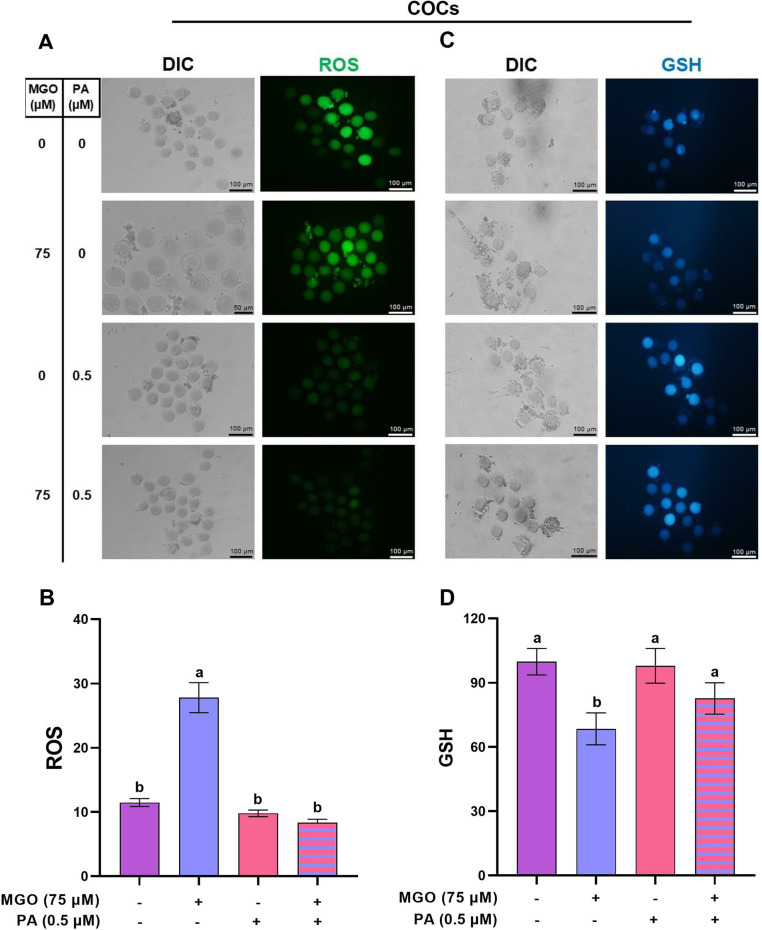
The optimal concentration of PA modulates the redox state in COCs challenged with MGO during IVM. COCs were recovered from ovaries of PMSG-stimulated NMRI mice were cultured in IVM medium with indicated concentrations of MGO (75 µM) and/or PA (0.5 µM) for 18 h, and subsequently were denuded for assessment of ROS levels (A and B) and GSH levels (C and D). The representative images showed the ROS and GSH levels using a fluorescence microscope. Image J was used to measure the fluorescence intensity of ROS and GSH levels. At least three replicates were included for each assessment and at least 10 oocytes were assessed in each replicate. Data are presented as means ±  SEM. Different letters (a and b) indicate significant differences between the treatment groups at *P* < 0.05. Statistical differences between groups were assessed using one-way ANOVA with LSD post-hoc test.

Furthermore, the mRNA expression of *Glo1*, *Rage*, *Nrf2,* and *Nf-κβ* genes were analyzed. Subsequently, the developmental competence of treated COCs across various groups was assessed in terms of maturation rate, 2PN rate and blastocyst rate. Finally, the quality of derived blastocysts was determined in terms of their allocation to inner cell mass (ICM) and trophectoderm (TE) lineages (Figs 4, 6, 8, 9A-C, and 10).

#### Experiment 4:

**Fig 5:**
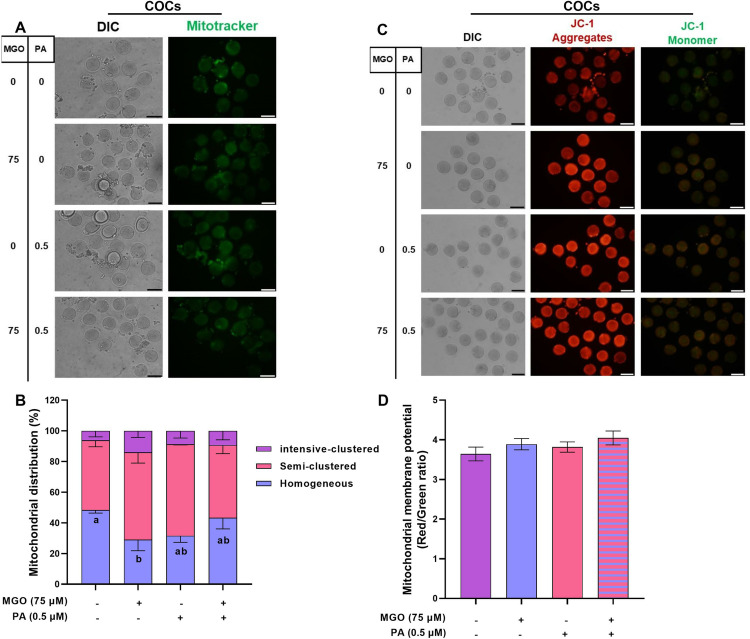
The optimal concentration of PA modulates the mitochondrial distribution but not mitochondrial membrane potential in COCs challenged with MGO during IVM. COCs were recovered from ovaries of PMSG-stimulated NMRI mice were cultured in IVM medium with indicated concentrations of MGO (75 µM) and/or PA (0.5 µM) for 18 h, and subsequently were denuded for assessment of mitochondrial distribution (A and B) and mitochondrial membrane potential (C and D). The representative images showed the mitochondrial distribution and mitochondrial membrane potential using a fluorescence microscope. Image J was used to measure the red/green fluorescence intensity. At least three replicates were included for each assessment and at least 10 oocytes were assessed in each replicate. Data are presented as means ±  SEM. Different letters (a and b) indicate significant differences between the treatment groups at *P* < 0.05. Statistical differences between groups were assessed using one-way ANOVA with LSD post-hoc test. The bars represent 50 µm.

Due to existing background and knowledge about the supportive and protective role of cumulus cells for oocytes, we designed this experiment to assess how PA could alleviate the adverse effects of MGO in DOs. The evaluation criteria mirrored those of Experiment 3 except for mRNA gene expression and differential staining of derived blastocysts. (Figs 5, 7, 9D-F, S1-S3).

### Collection of oocytes

Following the intraperitoneal injection of PMSG, mice were sacrificed by cervical dislocation 48 hours later. Ovary recovery and GV oocyte washing medium was HTCM (Sigma, USA, M5017) 10% FBS (Gibco, USA, 10270-110). Ovaries were excised, and both COCs and DOs containing fully grown GV oocytes were obtained by gently puncturing visible large antral follicles present on the ovary surface. Healthy GV oocytes were pooled and randomized before distribution into the experimental groups [25]. To study the developmental competence of oocytes under our experimental treatments, GV oocytes were placed into the IVM medium in the presence or absence of Methylglyoxal (Sigma, USA, M0252) and/or Punicic acid (Larodan AB, Sweden, 10-1875).

### In vitro maturation (IVM)

For oocyte maturation, drops (each ~20 µl) of maturation medium (α-MEM medium (Sigma, USA, M0644) supplemented with 5% FBS, 75 IU/ml HCG (Karma, Germany), and 0.1 IU/ml recombinant human FSH (Cinnal-f®, Cinnagen, Iran)) containing 5 COCs or DOs were incubated under oil in 35 mm culture dishes. The maturation rate was assessed following treatment with or without various concentrations of MG and/or PA for 18 h under an atmosphere of 6% CO_2_ at 37 °C in the incubator [26]. Maturation rate was reported at 18 h after IVM by observation of polar body.

### In vitro fertilization (IVF)

After approximately 18 hours of IVM, sperm were collected from adult NMRI male mice (10-12-weeks old) by puncturing the cauda epididymides with a needle. The collected sperm were then stored in Sperm Active Medium (Genocell Co., Iran, GC302) for 45 minutes for swmming up and capacitaion. Following this incubation, motility was assessed, and sperm concentration was determined using a Makler counting chamber. Approximately 1–2 million motile sperm per ml were added to each cumulus oocyte complex in drops of fertilization medium (G-IVF^TM^ PLUS Vitrolife Co., Sweden, 26078-09), allowing fertilization to occure over a 6 h period. Subsequently, the zygotes were washed in HTCM containing 10% FBS. They were then cultured in G-TL™ medium (G-TL^TM^ Vitrolife Co., Sweden, 26283-09) and incubated for approximately 4 days in an atmosphere of 5% O_2_ and 6% CO_2_ at 37 °C, until they reached the blastocyst stage [26].

### Differential staining

Differential staining was performed to identify theinner cell mass (ICM) and trophectoderm (TE) cells. Day 4 blastocysts were collected and washed three times in PBS (Gibco, USA, 21600-051) + PVA (Sigma, USA, P8136) and permeabilized using 0.5% triton‐X‐100 (Merck, Germany, 1086031000) in HTCM containing 5 mg/ml bovine serum albumin, for 30 s. Then, blastocysts were stained with 30 μg/ml propidium iodide (Sigma, USA, P4170) for 10 s. Subsequently, blastocysts were incubated with 10 µg/ml Hoechst 33342 (Invitrogen, USA, R37165) at 4°C for 15 min. Finally, blastocysts were mounted in light diagnostic mounting fluid (Merck, Germany, 5013) and observed under a fluorescence microscope(Olympus, IX71, Japan) equipped with DP72 camera (Olympus, Japan) using DP2‐BSW software. ICM and TE were recognized based on their blue and pinkcolors, respectively. Finally, total cell numbers (TCN: ICM +  TE) were calculated.

### Evaluation of mitochondrial distribution

Mito Tracker Green FM (Invitrogen, USA, M7514) was used for assessing mitochondrial distribution [27]. Briefly, MII oocytes were denuded by pipetting in hyaluronidase (80 IU/ml) and subsequently, incubated in phosphate-buffered saline without calcium and magnesium (PBS^-^) containing 400 nM Mito Tracker Green FM for about 30 min at 37°C. Then, they were washed and transferred to drops of PBS^-^ and images were recorded using an inverted fluorescent microscope equipped with a 520 nm emission filter. To analyze the mitochondrial distribution in MII oocytes, the oocytes were classified into 3 categories: homogenous, semi-clustered, and clustered [28].

### Evaluation of mitochondrial membrane potential

Mitochondrial membrane potential was measured by JC-1 (Abcam, USA, 113850) which is a lipophilic cationic dye. If the membrane potential is high, stained particles form J-aggregate and reflect red fluorescent and when the potential is low, they stay as monomers and reflect green fluorescent. The mitochondrial membrane potential was assessed by measuring the red to green fluorescence ratio in each individual oocyte. To this end, MII denuded oocytes (n =  35 to 40 oocytes per group in each replicate) were exposed to the JC-1 probe at a concentration of 20 μg/ml for 30 min at 37°C. After being washed with PBS^-^/PVA, oocytes were placed in groups of 10 in 20 µl drops of PBS^-^/PVA and observed under inverted fluorescence microscopy. Relative quantification of fluorescence intensity was performed by Image J software (National Institutes of Health, Bethesda, MD), as described previously [29].

### Measurement of ROS and GSH levels

Levels of ROS and GSH were measured according to previous studies [28,30]. Following the maturation of COCs, in various treatment groups, matured COCs were denuded. Subsequently, denuded matured oocytes were exposed to 10 μM 2, 7- dichloro dihydroflouresce in diacetate H2DCFA (Sigma, USA, D6883) for 30 min and 10 μM Cell Tracker Blue CMF2HC (Invitrogen™, USA, C12881) for 1 h at 37°C in the dark, to determine ROS and GSH levels, respectively. After washing the stained oocytes, they were placed into 10 μl drops of PBS/PVA and observed using an inverted fluorescent microscope. Immediately after exposure, a digital image of each matured oocyte was taken by a fluorescence camera. The fluorescence intensity of oocytes was analyzed using the ImageJ software (National Institutes of Health, Bethesda, MD). Assessment of ROS and GSH levels was conducted in three replicates, with a minimum of 30 matured COCs included in each group for each replicate.

### Evaluation of COCs mRNA expression

The mRNA expression of *Glo1*, *Rage*, *Nf-κβ*, and *Nrf2* genes were analyzed using real-time reverse transcription polymerase chain reaction (RT-PCR). The total RNA of 80 matured COCs in each replicate was extracted using RNeasy Micro Kit (QIAGEN, Germany, 74004) for each replicate according to the manufacturer’s protocol. Three independent biological replicates were conducted. Total RNA was reverse transcribed using a cDNA Synthesis kit (Biotechrabbit, Germany, BR0400403) in accordance with the manufacturer’s protocol. Quality and integrity of cDNA was checked using PCR and housekeeping primer (*Gapdh*), as a reference gene in the RT-PCR analyses. Three technical replicates were performed for each sample and the mean cycle threshold (Ct) was calculated [31]. Relative expression was computed using Ct values that were normalized against *Gapdh*. Fold change in gene expression was calculated using 2^-ΔCT^. All the primers were designed by the Primer 3 program (http://primer3.ut.ee/) and their characteristics are listed in the supplementary information (Table S1).

### Statistical data analysis

All assessments were performed at least three times and Data are presented as mean ±  S.E.M. Statistical significance was defined as *P* < 0.05. One-way analyses of variance (ANOVA) were applied to compare the effect of the treatments between the various groups (α =  0.05), followed by the LSD post-hoc test. Graphs were created using GraphPad Prism (v.6.0.1),while statistical analysis were performed using IBM SPSS program (v.23, NY, USA).

## Results

### MGO impaired maturation rate and developmental competence of COCs and DOs

To assess the impact of MGO on the developmental competence of matured oocytes, both COCs and DOs were subjected to different concentrations of MGO (0, 20, 40, 75, and 150 µM) for 18 hours during the maturation period.

Fig 1A and 1D demonstrate a significant decrease (*P* < 0.05) in the maturation rate of both COCs and DOs treated with 75 and 150 µM MGO in comparison to the other concentrations (0, 20, and 40 µM MGO). Interestingly, in DOs, the maturation rate was significantly lower (*P* < 0.05) in the 150 µM MGO group (13.77 ±  2.49) compared to the 75 µM MGO group (36.56 ±  0.73). Subsequently, the matured oocytes from both COCs and DOs underwent in vitro fertilization, and the rates of pronucleus formation and blastocyst development were evaluated.

In the COCs category, the pronucleus formation rate was significantly reduced (*P* < 0.05) in the 150 µM MGO group (33.49 ±  2.70) compared to the other treatment groups. Additionally, both 75 µM (3.09 ±  0.77) and 150 µM MGO (4.87 ±  2.88) significantly decreased (*P* < 0.05) the blastocyst rate compared to the other groups.

Notably, the impact of MGO on the developmental competence of matured oocytes was more pronounced in the DOs category (Fig S1). Both 75 µM (20.78 ±  3.82) and 150 µM MGO (2.84 ±  0.38) significantly decreased the pronucleus formation rate compared to the other groups in DOs. Intriguingly, all concentrations of MGO significantly diminished the blastocyst rate compared to the control group. Furthermore, when we compared the developmental competence of DOs to COCs exposed to varying concentrations of MGO, we observed that all developmental aspects of treated oocytes were more diminished in DOs (Fig S1). These findings emphasize the importance of cumulus cells in preserving the developmental competence of matured oocytes.

### Optimizing PA concentration: effects on maturation rate and developmental competence of COCs and DOs

PA, a major component of pomegranate seed oil, is known for its antioxidant properties. In light of its antioxidant effects, our objective was to investigate the impact of PA during IVM on the maturation rate and developmental competence of COCs and DOs.

To achieve this, GV oocytes (COCs) were cultured for 18 hours in maturation medium either in the absence of PA (Control) or in the presence of various concentrations of PA (0.5, 1, 10, 100, and 200 µM) and subsequently IVF was carried out.

Fig 2A illustrates that PA did not significantly affect the maturation rate of COCs across different PA concentrations that were used (*P* > 0.05). However, 200 µM PA (64.24 ±  5.85) significantly decreased the pronucleus formation rate and blastocyst rate (3.05 ±  0.79) of COCs compared to the other groups (*P* < 0.05, Fig 2B and 2C).

Furthermore, we investigated the impact of different concentrations of PA on the maturation rate and developmental competence of denuded GV oocytes. Interestingly, we observed that 0.5 µM PA (68.36 ±  3.29) increased the proportion of MII oocytes (*P* < 0.05), whereas other concentrations (including 0.25, 1, 2.5, 5, 10, and 80 µM PA) had no significant effect on the maturation rate compared to either the control or the 0.5 µM PA group (Fig 2D).

The pronucleus formation rate was significantly higher (*P* < 0.05) in the 0.5 µM PA group (54.71 ±  3.47) compared to the control (39.49 ±  2.83) and 80 µM PA groups (25.38 ±  3.97) (Fig 2E). It is worth noting that the pronucleus formation rate in the 80 µM PA group was significantly lower (*P* < 0.05) than inother treatment groups, except for the control group.

Furthermore, as shown in Fig 2F, the blastocyst formation rate was significantly higher (*P* < 0.05) in the 0.5 µM PA group (43.35 ±  10.51) compared to all other treatment groups. Additionally, the 80 µM PA group (0.52 ±  0.52) exhibited a significantly reduced (*P* < 0.05) blastocyst rate compared to all other treatment groups, except for the 5 and 10 µM PA groups (10.40 ±  4.97 and 9.44 ±  3.73, respectively).

To investigate the decreased developmental competence observed in COCs and DOs treated with high concentrations of PA (200 µM in COCs and 80 µM in DOs), we examined the levels of ROS and GSH. As shown in Fig 3, high concentrations of PA in both COCs (200 µM) and DOs (80 µM) resulted in a diminished developmental competence of matured oocytes, accompanied by an increase in ROS level (*P* < 0.05, Fig 3B and 3F) (14.83 ±  1.24 and 33.81 ±  2.41, respectively) and a reduction in GSH level (*P* < 0.05, Fig 3D and 3H) (27.98 ±  1.92 and 41.65 ±  4.67, respectively). Interestingly, our findings revealed that 0.5 µM PA had no significant impact on the ROS and GSH levels in matured COCs (9.13 ±  0.79 and 63.42 ±  2.59, respectively). However, in DOs, it led to a reduction (*P* < 0.05, Fig 3F and 3H) in ROS level (14.39 ±  1.14) and an increase in GSH level (70.25 ±  3.84). Furthermore, our data revealed that DOs either in the absence (control) or presence (0.5 µ M) of PA exhibited higher levels of ROS as compared to COCs which highlights the protective role of cumulus cells against oxidative stress (Fig S2).

### Protective effect of PA on COCs quality against MGO

Based on previously published studies, MGO has been shown to induce oxidative stress and negatively impact the quality of oocytes during IVM. In experiment 1, we selected 75 µM MGO as a damage mode. As demonstrated in Fig 4(A and B), the treatment with 75 µM MGO significantly increased (*P* < 0.05) the ROS levels in matured COCs (27.78 ±  2.34). Conversely, the level of GSH was remarkably decreased in matured COCs after exposure to 75 µM MGO (68.44 ±  7.43) during IVM (*P* < 0.05, Fig 4C-4D).

PA has recently been characterized for its antioxidant properties. To explore whether the impaired redox state could be restored in matured COCs using PA, we co-treated the MGO-challenged COCs and DOs with an optimized concentration of PA (0.5 µM) during IVM. Interestingly, we observed a significant reduction in ROS levels (8.35 ±  0.51) and an increase in GSH levels (82.67 ±  7.34) following the co-treatment with PA in comparison to the MGO-challenged COCs (*P* < 0.05, Fig 4). These values were similar to those observed in the control group (*P* > 0.05).

Furthermore, we examined whether the imbalanced redox state in MGO-challenged COCs could disrupt mitochondrial distribution and membrane potential. As shown in Fig 5(A and B), the proportion of COCs with homogeneous mitochondrial distribution significantly decreased (*P* < 0.05) in the presence of 75 µM MGO (29.03 ±  7.19). However, the co-treatment of MGO-challenged COCs with 0.5 µM PA reversed this perturbed mitochondrial distribution to a level similar to that of the control group (*P* > 0.05).

It is noteworthy to mention that the mitochondrial membrane potential, remained unchanged in matured COCs in the presence of MGO and in the co-treatment of MGO with PA (*P* > 0.05, Fig 5C-5D).

### Co-treatment of MGO-challenged COCs with PA during IVM modulates the mRNA expression of Glo1, Nrf2, Rage, and Nf-κβ genes

**Fig 6:**
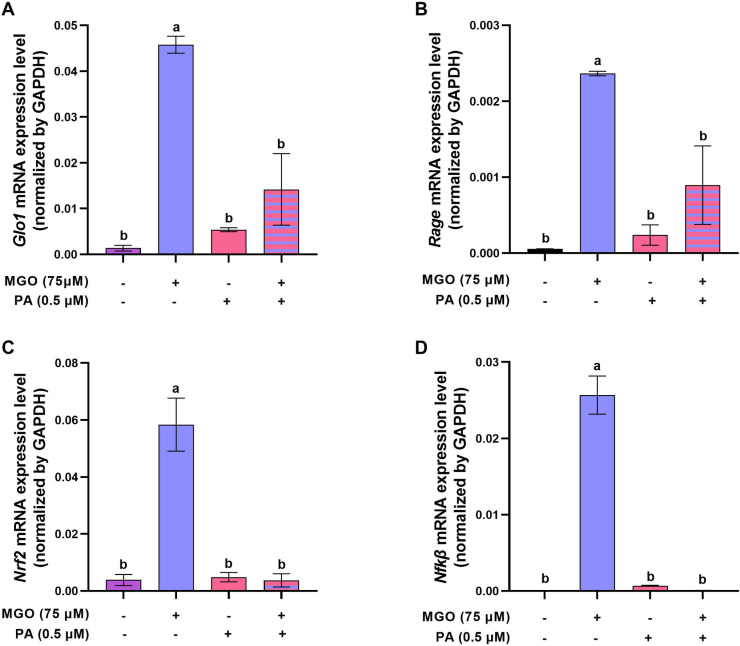
The optimal concentration of PA modulates the mRNA expression of *Glo1*, *Rage*, *Nrf2*, and *Nf-κβ* genes of MGO-challenged COCs. The relative mRNA expression of (A) *Glo1*, (B) *Rage*, (C) *Nrf2* and (D) *Nf-κβ* genes in COCs after treatment with MGO or/and 0.5 µ M of PA in IVM medium for 18 h. Each experiment was carried out at least in three independent replications and in each replication at least 80 COCs were used in each group. Data are presented as means ±  SEM. Different letters (a, b, c) indicate significant differences between the treatment groups at *P* < 0.05. Statistical differences between groups were assessed using one-way ANOVA with LSD post-hoc test.

GLO1 is an enzyme involved in the detoxification of MGO in various cells. Our data revealed that in the presence of 75 µM MGO during IVM, the mRNA expression of *Glo1* significantly increased in COCs (Fig 6A, *P* < 0.05). However, following co-treatment of COCs exposed to MGO stress with 0.5 µ M PA, this elevated expression level significantly decreased (Fig 6A, *P* < 0.05), resembling the control group (Fig 6A, *P* > 0.05).

Methylglyoxal can affect the expression of RAGE, a cell surface receptor involved in the signaling of AGEs. Previous studies have shown that exposure to methylglyoxal can increase the formation of AGEs, subsequently activating the RAGE signaling pathway. To assess the mRNA expression of *Rage*, a key player in the AGE/RAGE reaction, we found that similar to the pattern of *Glo1* expression, the mRNA expression of *Rage* increased in COCs challenged with MGO (Fig 8B, *P* < 0.05). Furthermore, co-treatment with PA rescued the mRNA expression of *Rage*, reducing its expression to a level similar to the control group (Fig 6B, *P* > 0.05).

NRF2 is a transcription factor involved in cellular defense mechanisms against oxidative stress and toxic insults. Additionally, NF-κβ is a transcription factor crucial in regulating immune responses and inflammatory processes. Therefore, we evaluated the mRNA expression of both *Nrf2* and *Nf-κβ* in the COCs of different treatment groups. As shown in Fig 6C and 6D, we observed a similar increase in mRNA expression of both *Nrf2* and *Nf-κβ* in MGO-treated COCs compared to the control group. However, co-treatment of MGO-exposed COCs with PA significantly reduced the mRNA expression of these two genes (Fig 6C and 6D, *P* < 0.05), making the mRNA levels similar to those of the control group (*P* > 0.05).

### PA can ameliorate the reduced developmental competence of MGO-challenged COCs

**Fig 7:**
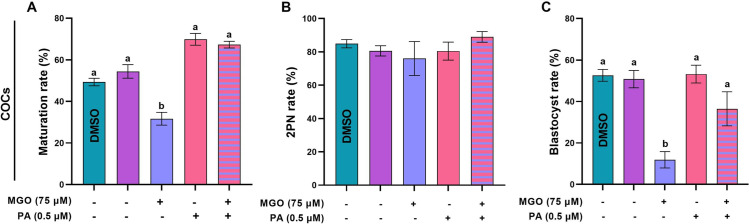
PA ameliorates the reduced developmental competence of MGO-challenged COCs. COCs and DOs were recovered from PMSG-stimulated NMRI mice and cultured in IVM medium with indicated concentrations of MGO (75 µM) and/or PA (0.5 µM) for 18 h and fertilized *in vitro*, and transferred to *in vitro* culture medium. A) maturation rates, B) pronucleus formation rates, and C) blastocyst rates in COCs, respectively. At least 40 COCs were cultured in each replicate of each treatment group. At least three replicates were included in each treatment group. Data are presented as means ±  SEM. Different letters (a, b, and c) indicate significant differences between the treatment groups at *P* < 0.05. Statistical differences between groups were assessed using one-way ANOVA with LSD post-hoc test.

To investigate whether PA could improve the reduced developmental competence of COCs treated with 75 µM MGO, we co-treated GV oocytes with 75 µM MGO and 0.5 µM PA during IVM. Our data revealed that supplementation of the IVM medium with 0.5 µM PA improved the maturation (67.29 ±  1.60) and blastocyst rates (36.46 ±  8.20) of MGO-challenged COCs (Fig 7A and 7C, *P* < 0.05). However, the 2PN formation rate remained unchanged in the presence of MGO and in the co-treatment of MGO with PA in matured COCs (Fig 7B, *P* > 0.05).

### PA restored impaired cell lineage allocation in blastocysts developed from MGO-challenged COCs

**Fig 8:**
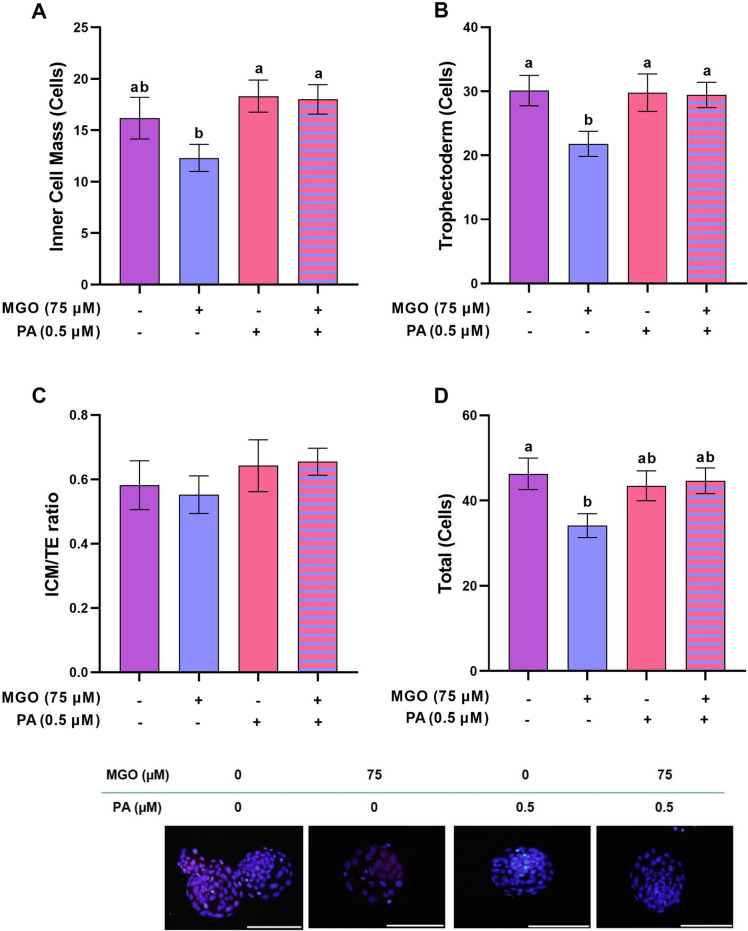
The optimal concentration of PA restores the impaired cell lineage allocation in blastocysts developed from MGO-challenged COCs. COCs were recovered from PMSG-stimulated NMRI mice and cultured in IVM medium with indicated concentrations of MGO (75 µM) and/or PA (0.5 µM) for 18 h and fertilized *in vitro*, and transferred to *in vitro* culture medium. After 4.5 day the derived blastocysts were differentially stained to be assessed for the number of A) inner cell mass (ICM), B) trophectoderm (TE), C) ICM/TE ration and D) Total cell numbers (TCN). E) Representative images show the ICM cells in blue and TE cells in pink. Number of stained blastocysts was 30 in each group. Data are presented as means ±  SEM. Different letters (a and b) indicate significant differences between the treatment groups at *P* < 0.05. Statistical differences between groups were assessed using one-way ANOVA with LSD post-hoc test.

Blastocysts developed from MGO-challenged COCs for 4 days had a reduced TE and TCN (Fig 8B and 8D, *P* < 0.05). However, the ICM and ICM/TE ratio in the 75 µM MGO group remained unchanged compared to the control group (Fig 8A and 8C, *P* > 0.05). Subsequently, we assessed if co-treatment with 0.5 µM PA improved cell lineage allocation in blastocyst from the MGO group. Interestingly, we found that co-treatment with 0.5 µM PA attenuated MGO impacts such that the ICM (Fig 8A) and TE (Fig 8B) cell numbers were significantly higher than MGO group (*P* < 0.05) and not significantly different from standard culture (*P* > 0.05). In addition, it is noteworthy that TCN in MGO+PA tends to be significantly higher (Fig 8D, *P* = 0.066) than MGO group.

### Treatment of MGO-challenged DOs with PA improves the quality of matured oocytes

**Fig 9:**
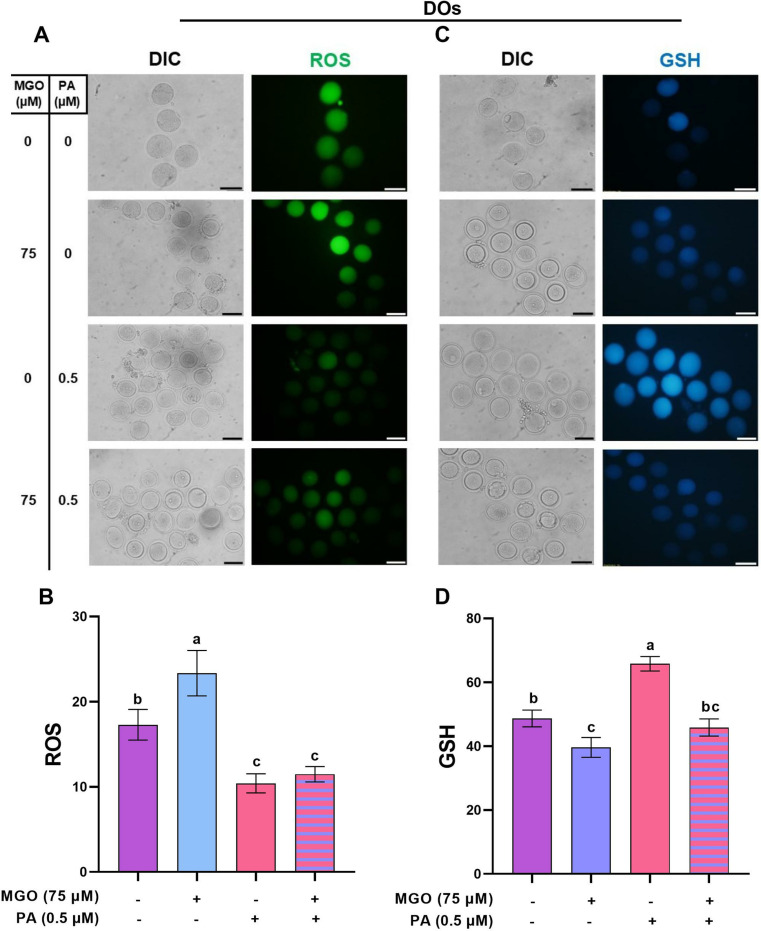
The optimal concentration of PA modulates the redox state in DOs challenged with MGO during IVM. DOs were recovered from ovaries of PMSG-stimulated NMRI mice were cultured in IVM medium with indicated concentrations of MGO (75 µM) and/or PA (0.5 µM) for 18 h assessment of ROS levels (A and B) and GSH levels (C and D). The representative images showed the ROS and GSH levels using a fluorescence microscope. Image J was used to measure the fluorescence intensity of ROS and GSH levels. At least three replicates were included for each assessment and at least 10 oocytes were assessed in each replicate. Data are presented as means ±  SEM. Different letters (a, b and c) indicate significant differences between the treatment groups at *P* < 0.05. Statistical differences between groups were assessed using one-way ANOVA with LSD post-hoc test. The bars represent 50 µm.

To investigate the role of cumulus cells and further examine how DOs respond to MGO during the maturation period, we designed experiment 4. As demonstrated in Fig 9(A and B), the treatment with 75 µM MGO significantly increased the ROS levels in matured DOs (23.35 ±  2.66, *P* < 0.05). Conversely, the level of GSH was remarkably decreased in matured DOs after exposure to 75 µM MGO (39.60 ±  3.09) during IVM (Fig 9C-9D, *P* < 0.05).

Following the co-treatment with PA, we observed a significant reduction in ROS levels (11.47 ±  0.90, *P* < 0.05) and a non-significant increase in GSH levels (45.84 ±  2.66, *P* > 0.05) in comparison to the MGO-challenged DOs (Fig 9). These values were similar to those observed in the control group.

**Fig 10:**
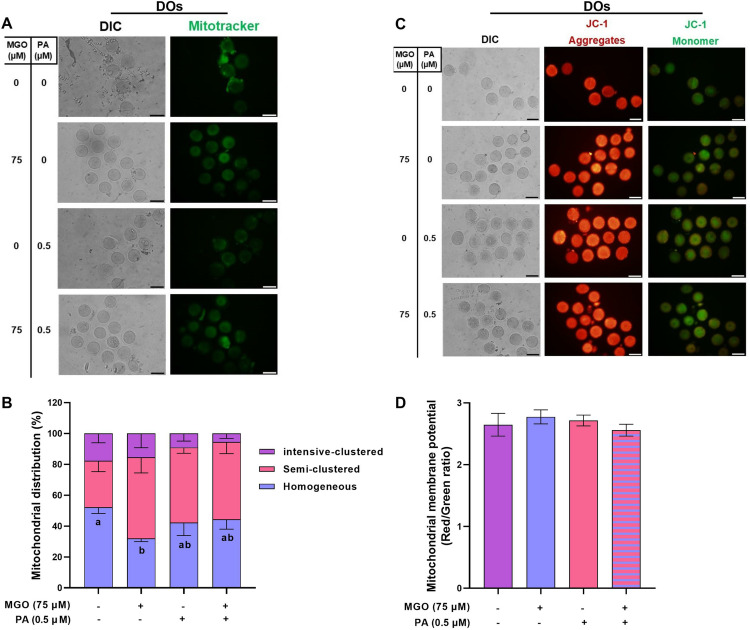
The optimal concentration of PA modulates the mitochondrial distribution but not mitochondrial membrane potential in DOs challenged with MGO during IVM. DOs were recovered from ovaries of PMSG-stimulated NMRI mice were cultured in IVM medium with indicated concentrations of MGO (75 µM) and/or PA (0.5 µM) for 18 h for assessment of mitochondrial distribution (A and B) and mitochondrial membrane potential (C and D). The representative images showed the mitochondrial distribution and mitochondrial membrane potential using a fluorescence microscope. Image J was used to measure the red/green fluorescence intensity. At least three replicates were included for each assessment and at least 10 oocytes were assessed in each replicate. Data are presented as means ±  SEM. Different letters (a and b) indicate significant differences between the treatment groups at *P* < 0.05. Statistical differences between groups were assessed using one-way ANOVA with LSD post-hoc test. The bars represent 50 µm.

Furthermore, we examined the mitochondrial distribution and membrane potential MGO-challenged DOs. As shown in Fig 10(A and B), the proportion of DOs with homogeneous mitochondrial distribution significantly decreased (*P* < 0.05) in the presence of MGO (32.03 ±  2.06). However, the co-treatment of MGO-challenged DOs with PA reversed this perturbed mitochondrial distribution to a level similar to that of the control group (*P* > 0.05). Finally, the mitochondrial membrane potential remained unchanged in matured DOs in the presence of MGO and in the co-treatment of MGO with PA (*P* > 0.05, Fig 10C-10D).

### PA can ameliorate the reduced developmental competence of MGO-challenged DOs

**Fig 11:**
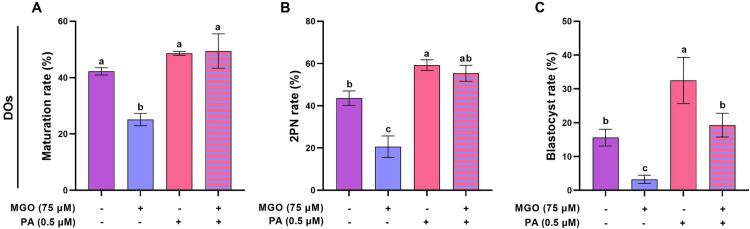
PA ameliorates the reduced developmental competence of MGO-challenged DOs. DOs were recovered from PMSG-stimulated NMRI mice and cultured in IVM medium with indicated concentrations of MGO (75 µM) and/or PA (0.5 µM) for 18 h and fertilized *in vitro*, and transferred to *in vitro* culture medium. A) maturation rates, B) pronucleus formation rates, and C) blastocyst rates in DOs, respectively. At least 30 DOs were cultured in each replicate of each treatment group. At least three replicates were included in each treatment group. Data are presented as means ±  SEM. Different letters (a, b, and c) indicate significant differences between the treatment groups at *P* < 0.05. Statistical differences between groups were assessed using one-way ANOVA with LSD post-hoc test.

The effect of PA on ameliorating the perturbed developmental competence of DOs that were exposed to MGO were assessed in terms of maturation, 2PN formation, and blastocyst rates up to 4.5 days post-insemination. Our data revealed that supplementation of the IVM medium with 0.5 µM PA improved the maturation, 2PN formation, and blastocyst rates of MGO-challenged DOs (Fig 11A and 11C, *P* < 0.05).

Finally, our data (Figs S1 and S3) indicate that, irrespective of the treatment groups, DOs faced more significant challenges against MGO during the maturation period, resulting in lower developmental competence thanCOCs. This observation further underscores the protective role of cumulus cells in safeguarding oocytes within a pathophysiological microenvironment. Notably, treatment with 0.5 µM PA exhibited similar beneficial effects on both MGO-challenged COCs and DOs, enhancing their developmental competence to a comparable level. These findings suggest that PA may serve as a potent and promising antioxidant for future investigations.

## Discussion

The prevalence of hyperglycemia among women of childbearing age worldwide poses significant challenges to fertility. Concurrently, dietary factors have been implicated in affecting fertility in both animals and humans. One crucial aspect is the increase in levels of MGO induced by both hyperglycemia and malnutrition. Limited studies have demonstrated the detrimental impact of AGEs on various aspects of pre-implantation development potentially, compromising embryo quality and successful implantation. To address this, recent studies have explored integrating nutritional interventions with metabolic disorders to mitigate their adverse effects on reproductive health.

In line with this approach, our current study focuses on alleviating the adverse effects of MGO during IVM by supplementing the IVM medium with punicic acid, a novel conjugated polyunsaturated fatty acid (PUFA), in a mouse model. Furthermore, given the protective role of cumulus cells against oxidative stressors, including MGO, our study aims to investigate their distinct impact on oxidative stress within cumulus-oocyte complexes (COCs) compared to denuded oocytes (DOs) during IVM, shedding light on the protective mechanisms of cumulus cells against MGO-induced stress [32].

Our data demonstrate significant impacts of MGO exposure during IVM on various developmental aspects of COCs and DOs. Specifically, exposure to 75 and 150 µM MGO decreased maturation and pronucleus formation rates in both COCs and DOs while also reducing blastocyst rates, particularly in DOs. However, co-treatment with 0.5 µM PA effectively restored the redox state and mitochondrial distribution in both COCs and DOs, leading to improved maturation and blastocyst rates in MGO-challenged oocytes. Furthermore, our study revealed a significant downregulation of *Glo1*, *Rage*, *Nrf2*, and *Nf-κβ* genes in MGO-exposed COCs, which was attenuated by PA co-treatment, suggesting a potential mechanism underlying the protective effects of PA.

These findings are consistent with earlier studies by Edmidio et al., Liu et al., and Chang et al., all of which similarly reported adverse effects of MGO on oocyte maturation and developmental competence. However, our study expands upon these findings by elucidating the specific impacts on mitochondrial distribution and gene expression, as well as the protective effects co-treatment with PA. Overall, our results underscore the importance of mitigating MGO-induced oxidative stress during IVM to enhance oocyte quality and developmental competence [25,26,33].

In our study, we observed a maturation rate of approximately 60% in the control group, which is lower than the rates typically reported in other studies in mice species, where maturation rates often reach around 90%. To investigate this discrepancy, we conducted additional experiments to assess polar body extrusion at different time points after IVM. Surprisingly, we found that 92.5% of oocytes extruded the first polar body (FPB) at 12 hours post-IVM, followed by a decrease to around 75% at 15 hours and further decline to 60% at 18 hours. This observation suggests a time-dependent degeneration of some FPBs following nuclear maturation, consistent with findings by Miao et al [34].

Our results showed that 75 and 150 µM MGO concentrations significantly reduced the blastocyst rate in COCs. However, all tested concentrations of MGO (20, 40, 75, and 150 µM) significantly reduced the blastocyst rate in DOs. This observation underscores the protective role of cumulus cells, which provide essential nutrients, growth factors, and regulatory molecules to support oocyte maturation and developmental potential [32–38]. Cumulus cells play a crucial role in the removal of harmful substances and the maintenance of a stable microenvironment surrounding the oocyte [37,38]. In a study by Tatone et al., the presence of *Glo1* and *Glo2*, two specialized detoxifying enzymes responsible for intracellular methylglyoxal detoxification, was identified in both oocytes and cumulus cells. Notably, the research revealed a significant decrease in the mRNA levels of *Glo1* and *Glo2* during the *in vivo* maturation of oocytes (from GV to MII) [36]. This suggests that the absence of cumulus cells in DOs, along with the expression of GLO1 and GLO2 in cumulus cells, may result in a reduced capacity to detoxify MGO compared to COCs. Consequently, DOs exhibit lower developmental competence than COCs when exposed to MGO.

PA, a bioactive compound in pomegranate seed oil, has been reported to have antioxidant and anti-inflammatory properties in various biological systems [39–41]. However, its effects on oocyte or embryo development remain largely unexplored. only a limited number of studies have assessed the effects of unconjugated linolenic acid on the developmental competence of oocytes or embryos in *in vitro* conditions [42,43]. In our study, treatment with 0.5 µM PA improved blastocyst rates in DOs, potentially due to its antioxidant effects, as evidenced by decreased ROS levels. However, PA did not significantly affect blastocyst rates in COCs, suggesting differences in the susceptibility to oxidative stress between cumulus-free oocytes and those surrounded by cumulus cells.

In addition, we observed that treatment of COCs and DOs with high concentrations of PA during IVM significantly reduced blastocyst rates. One possible reason for the decrease in blastocyst rate with a high concentration of PA is its influence on redox state and apoptosis. It has been shown that “reductive stress” similar to “oxidative stress” increases the ROS level [44]. Concerning this, we assessed the levels of ROS and GSH in treated COCs and DOs exposed to high concentrations of PA (200 µM and 80 µM, respectively). We observed that excessive levels of PA induce oxidative stress within the oocytes, leading to increased levels of ROS and decreased levels of GSH. Therefore, the toxic effect may be due to toxic concentrations rather than PA-inducing reductive stress. This oxidative stress can disrupt critical cellular processes, including DNA integrity, mitochondrial function, and protein synthesis, ultimately affecting the developmental competence of the oocytes [45].

Previous studies have elucidated how MGO and AGEs induce oxidative stress and inflammatory reactions through interaction with the RAGE, leading to increased transcription of inflammatory genes and apoptosis [46]. In our study, we observed that 75 µM MGO increased ROS levels and decreased GSH levels in both COCs and DOs. However, supplementation with 0.5 µM PA rectified the perturbed redox balance in MGO-challenged oocytes, restoring ROS and GSH levels to those of the control group. Supporting our findings, Marei et al. demonstrated that adding unconjugated linolenic acid to the IVM medium diminished elevated ROS levels similarly to the control group [47]. Additionally, studies have confirmed the antioxidant effects of unconjugated and conjugated linolenic acid in various oxidative stress conditions [48]. Istifli et al. showed the antioxidative effects of α-linolenic acid in cisplatin-induced nephrotoxicity by elevating the activity of CAT, SOD, and GPx enzymes in the renal tissue [49]. At the same time Anusree et al. demonstrated the reduction of ROS accumulation by PA supplementation in TNF-α-treated cells [41]. Furthermore, Saha et al. demonstrated the synergistic antioxidant effect of α-eleostearic acid and punicic acid against sodium arsenite-induced oxidative stress [50]. These studies collectively suggest that PA, due to its double bonds, possesses potent antioxidant properties, scavenging free radicals and reducing the formation of hydroperoxides, thus acting as a chain-breaking antioxidant by trapping chain-propagating free radicals.

In our study, we observed elevated levels of ROS and reduced levels of GSH in both COCs and DOs challenged with MGO. GSH content indicates proper cytoplasmic maturation in oocytes for progressive preimplantation embryonic development [43,44,51]. MGO can directly react with GSH, leading to the formation of a stable adduct called hemithioacetal [52], thereby depleting GSH levels consistent with our results that we observed higher expression of *Glo1* and reduced level of GSH in MGO-challenged COCs. Co-treatment of MGO-challenged oocytes with PA normalized GSH levels, as supported by previous studies demonstrating the restoration of GSH following PA administration. In agreement with our study, few studies demonstrated the restoration of GSH following the administration of PA. Concerning this, Waly et al. used an AOM-induced tumor model in Sprague-Dawley rats and they found that pomegranate peel extract can increase total antioxidant concentration (TAC), elevation in GSH, GST, GPx, glutathione reductase (GR), SOD and CAT [53]. The same group also reported that administration of pomegranate peel extract elevated the GSH/GSSG ratio [54]. Hassan et al. showed the positive effect of α-linolenic acid on oxidative stress by normalization of intracellular GSH concentrations in Rats with TNBS-Induced Colitis [55].

The redox state plays a crucial role in mitochondrial function. It is well demonstrated that MGO affects mitochondrial proteins and functions [56], however, very limited studies verified its effect on oocytes and preimplantation embryos on mitochondrial function. We observed perturbed mitochondrial distribution in MGO-challenged COCs and DOs, consistent with previous findings. Tatone and colleagues revealed that exposure of mouse oocytes to MGO perturbed mitochondrial distribution [36]. Furthermore, Luo et al. found that MGO-induced DNA damage and mitochondrial dysfunction fail porcine oocyte maturation and low *in vitro* development capacity of parthenogenetic and IVF embryos [57,58]. Our data revealed that PA reversed the mitochondrial perturbation, suggesting a role in modulating mitochondrial metabolism. To our knowledge, no studies have investigatedthe use of PA under either normal *in vitro* conditions or challenged conditions in oocytes and/or embryos. Nevertheless, very limited studies have assessed the effect of unconjugated linolenic acid on oocytes. Marei et al. showed that ALA abolished the reduction in mitochondrial membrane potential in the CCs of COCs matured under lipotoxic *in vitro* conditions [47]. While further research is needed to fully understand the specific mechanisms of PA involved in mitochondrial regulation, our study contributes to the growing body of evidence supporting the antioxidant activity of PA in oocytes.

Our study observed an increase in *Glo1* mRNA expression in the presence of MGO, suggesting an adaptive response to enhance the glyoxalase system’s detoxification capacity. Conversely, co-treatment of MGO-challenged COCs with PA decreased *Glo1* mRNA expression. Previous research by Liu et al. demonstrated that pomegranate phenolics inhibit AGE formation by scavenging reactive carbonyl species, suggesting a potential interaction between PA and pathways influenced by MGO exposure. These interactions may contribute to a complex molecular interplay ultimately downregulating Glo1 mRNA expression.

Previous studies have established a relationship between the AGE-RAGE system and infertility [35,59–61], providing a foundation for our investigation. In our study, we observed an increase in the mRNA expression of *Rage* in COCs challenged with MGO, which subsequently decreased in the presence of PA. This finding aligns with previous research by Diamanti-Kandarakis et al., who demonstrated increased AGEs and RAGE expression in ovarian tissue samples from women with polycystic ovary syndrome (PCOS) compared to controls [12]. Additionally, this group showed that women with PCOS had increased RAGE expression in monocytes compared to controls.

Furthermore, our study revealed an impaired redox state and increased mRNA expression of *Nrf2* and *Nf-κβ* in MGO-challenged COCs. In response to oxidative stress, NRF2 is a key transcription factor that plays a critical role in cellular defense mechanisms. Through the translocation of cytoplasm to the nucleus, NRF2 binds to antioxidant response elements (AREs) present in the promoter regions of various genes. This binding initiates the transcription of various antioxidant and detoxification genes. These genes encode proteins involved in scavenging ROS, restoring redox balance, and enhancing cellular defense against oxidative stress [62,63].

Similarly, NF-κβ activation by MGO contributes to inflammation and upregulation of RAGE expression in oocytes [64,65]). The presence of AGEs can activate RAGE, leading to a positive feedback loop where RAGE expression is further upregulated [66,67]. Our data verifies these possibilities.

Co-treatment of MGO-challenged oocytes with PA decreased the mRNA expression of *Rage*, *Nrf2*, and *Nf-κβ*, consistent with its anti-inflammatory and antioxidant effects. Previous studies have demonstrated the anti-inflammatory effects of PA by modulating the NF-κβ signaling pathway and scavenging free radicals, thereby mitigating inflammation and oxidative stress.

Throughout oocyte maturation, fertilization, and embryonic development, complex interactions ensure proper embryonic development. However, exposure to a chemical injury can lead to developmental issues or malformations. In our study, we found that MGO in the maturation medium adversely affected the redox state and mitochondrial distribution in matured mouse oocytes. To assess how these changes impact developmental competence, we conducted IVF followed by IVC in matured oocytes.

Our data revealed that MGO significantly reduced blastocyst formation, corroborating findings by Chang and colleagues, who reported that MGO inhibits mouse oocyte maturation, and embryonic development, inducing apoptosis and impairing blastocyst development. We also investigated the potential protective effects of PA against MGO-induced damage. Our results indicated that PA supplementation in the IVM medium significantly enhanced all aspects of developmental competence in matured oocytes.

Supporting our findings, limited studies suggest that AGE accumulation in infertile patients may contribute to reduced folliculogenesis and poor oocyte quality [68,69]. Our study reinforces the negative impact of AGEs on reproductive outcomes and highlights the potential therapeutic benefits of natural antioxidants like PA in mitigating AGE-induced damage.

## Conclusion

In conclusion, our study provides novel insights into the molecular mechanisms underlying MGO-induced damage in oocytes and the protective effects of PA against such damage. By exerting antioxidant effects, modulating the redox state, preserving mitochondrial distribution, and regulating gene expression, PA enhance the developmental competence of both MGO-challenged COCs and DOs. Further research is warranted to fully elucidate the therapeutic potential of PA in improving oocyte quality and reproductive outcomes in clinical settings.

## Supplementary information

S1 Table Primers used in this study for RT-PCR. (DOCX)

S1 Fig Comparing the developmental competence of COCs versus DOs recovered from PMSG-stimulated NMRI mice cultured in presence of various concentrations of MGO (0, 20, 40, 75 and 150 µM) during IVM: A) maturation rate, B) pronucleus formation and C) blastocyst rate. Data are presented as means ±  SEM. Asterisks demonstrate significant differences between groups, Statistical differences between groups were assessed using independent sample t-test. *  *P* < 0.05; ** *P* < 0.01 and *** *P* < 0.001.
(TIF)

S2 Fig Comparing A) ROS and B) GSH levels in COCs versus DOs recovered from PMSG-stimulated NMRI mice cultured in absence or presence of 0.5 µM PA during IVM. Data are presented as means ±  SEM. Asterisks demonstrate significant differences between groups, Statistical differences between groups were assessed using independent sample t-test. ** *P* < 0.01 and *** *P* < 0.001.
(TIF)

S3 Fig Comparing the developmental competence of COCs versus DOs recovered from PMSG-stimulated NMRI mice cultured in IVM medium with indicated concentrations of MGO (75 µM) and/or PA (0.5 µM) for 18 h: A) maturation rate, B) pronucleus formation rate and C) blastocyst rate. Data are presented as means ±  SEM. Asterisks demonstrate significant differences between groups, Statistical differences between groups were assessed using independent sample t-test. *  *P* < 0.05; ** *P* < 0.01 and *** *P* < 0.001.
(TIF)
