# Supplementary material for: Punicic acid alleviates methylglyoxal-induced oocyte dysfunction during in vitro maturation in mouse species
Source: PLoS One. 2025 Mar 25;20(3):e0314602. doi: 10.1371/journal.pone.0314602 (PMC11936299; doi:10.1371/journal.pone.0314602)
Supplement: S1 Table — (DOCX) [file pone.0314602.s001.docx]

| Gene name | Sequence | Product size | Annealing T | Accession number |
| --- | --- | --- | --- | --- |
| *Gapdh* | Forward 5`-TGCCGCCTGGAGAAACC-3`  Reverse 5`-TGAAGTCGCAGGAGACAACC-3` | 121 | 60 | [NM_001411843.1](https://www.ncbi.nlm.nih.gov/entrez/viewer.fcgi?db=nucleotide&id=2295558070) |
| *Glo1* | Forward 5`-ATCCAGACCCTAGCACCA-3`  Reverse 5`-TCAGTCCAAGAACCCTCGTA-3` | 102 | 60 | [NM_001113560.1](https://www.ncbi.nlm.nih.gov/entrez/viewer.fcgi?db=nucleotide&id=165932332) |
| *Nf- κβ* | Forward 5`-ACCGTAACAGCAGGACCCAAG-3`  Reverse 5`-AATACACGCCTCTGTCATCCG-3` | 117 | 60 | [NM_001410442.1](https://www.ncbi.nlm.nih.gov/entrez/viewer.fcgi?db=nucleotide&id=2283599767) |
| *Nrf2* | Forward 5`-GCTCTCCATATTCCATTCC-3`  Reverse 5`-TACCTCTCCTGCGTATATC-3` | 130 | 55 | [NM_010902.5](https://www.ncbi.nlm.nih.gov/entrez/viewer.fcgi?db=nucleotide&id=2199407475) |
| *Rage* | Forward 5`-CGAGGGAAGGAGGTCAAGTC-3`  Reverse 5`-GCCATCGGGAATCAGAAGT-3` | 192 | 54 | [NM_001271424.1](https://www.ncbi.nlm.nih.gov/entrez/viewer.fcgi?db=nucleotide&id=406855407) |
