# Supplementary figures and images for: Punicic acid alleviates methylglyoxal-induced oocyte dysfunction during in vitro maturation in mouse species
Source: PLoS One. 2025 Mar 25;20(3):e0314602. doi: 10.1371/journal.pone.0314602 (PMC11936299; doi:10.1371/journal.pone.0314602)

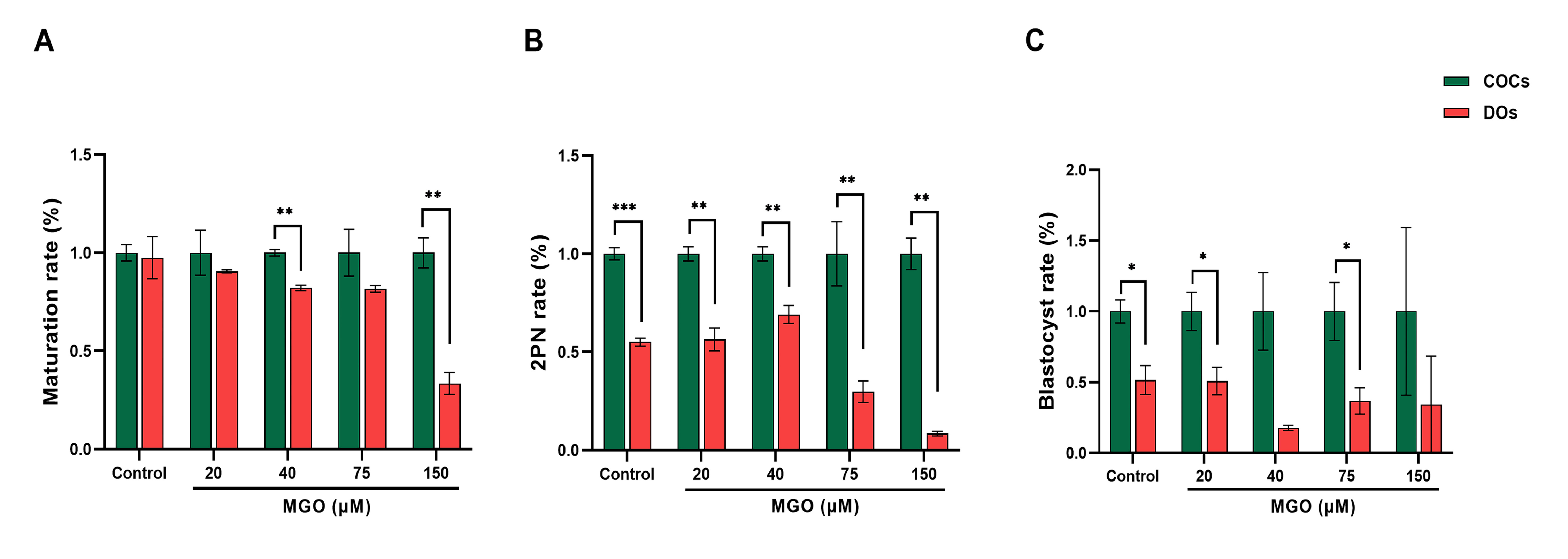

Supplement: S1 Fig — A) maturation rate, B) pronucleus formation and C) blastocyst rate. Data are presented as means ± SEM. Asterisks demonstrate significant differences between groups, Statistical differences between groups were assessed using independent sample t-test. * P < 0.05; ** P < 0.01 and *** P < 0.001. (TIF) [file pone.0314602.s002.tif]

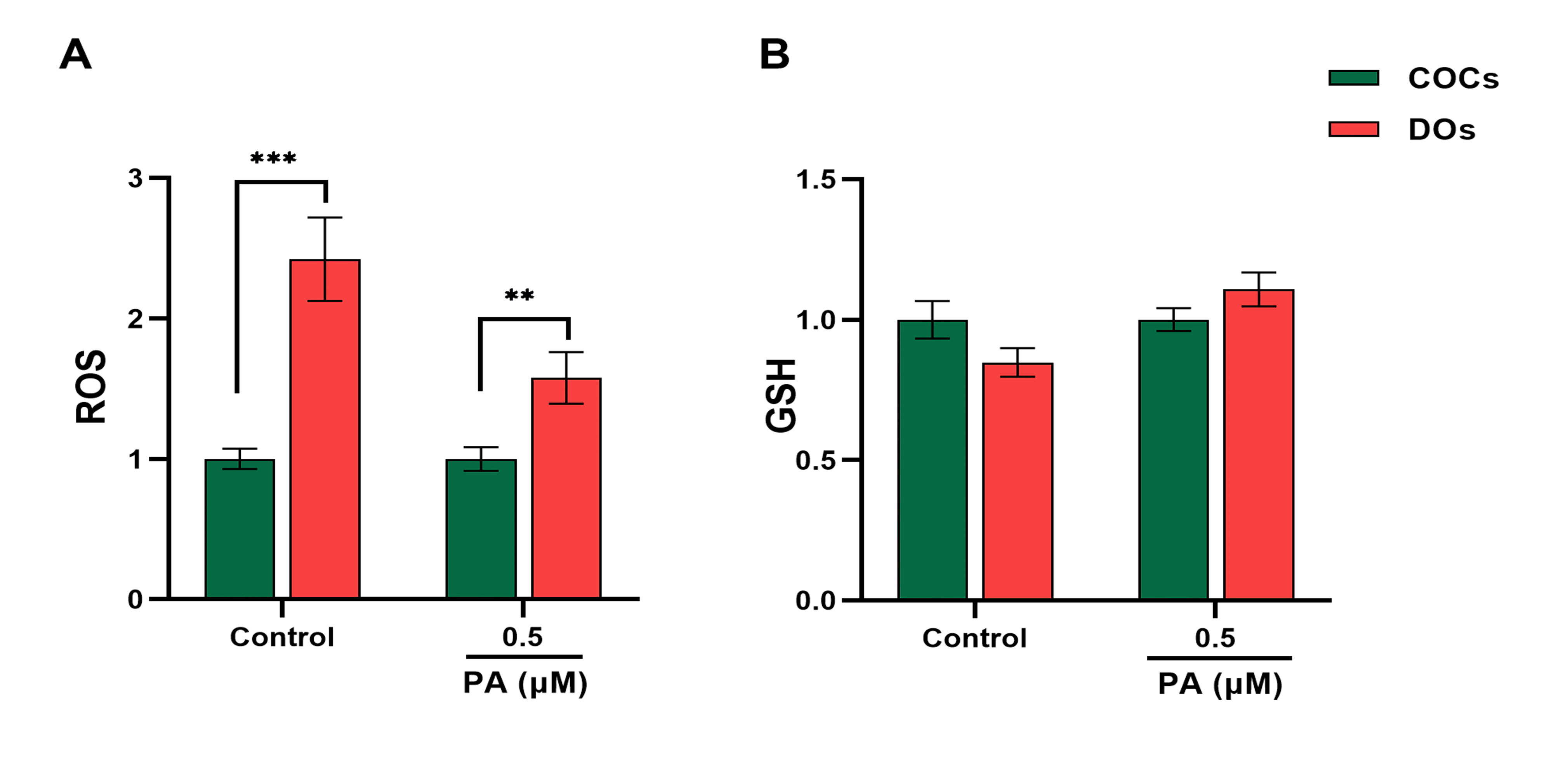

Supplement: S2 Fig — Asterisks demonstrate significant differences between groups, Statistical differences between groups were assessed using independent sample t-test. ** P < 0.01 and *** P < 0.001. (TIF) [file pone.0314602.s003.tif]

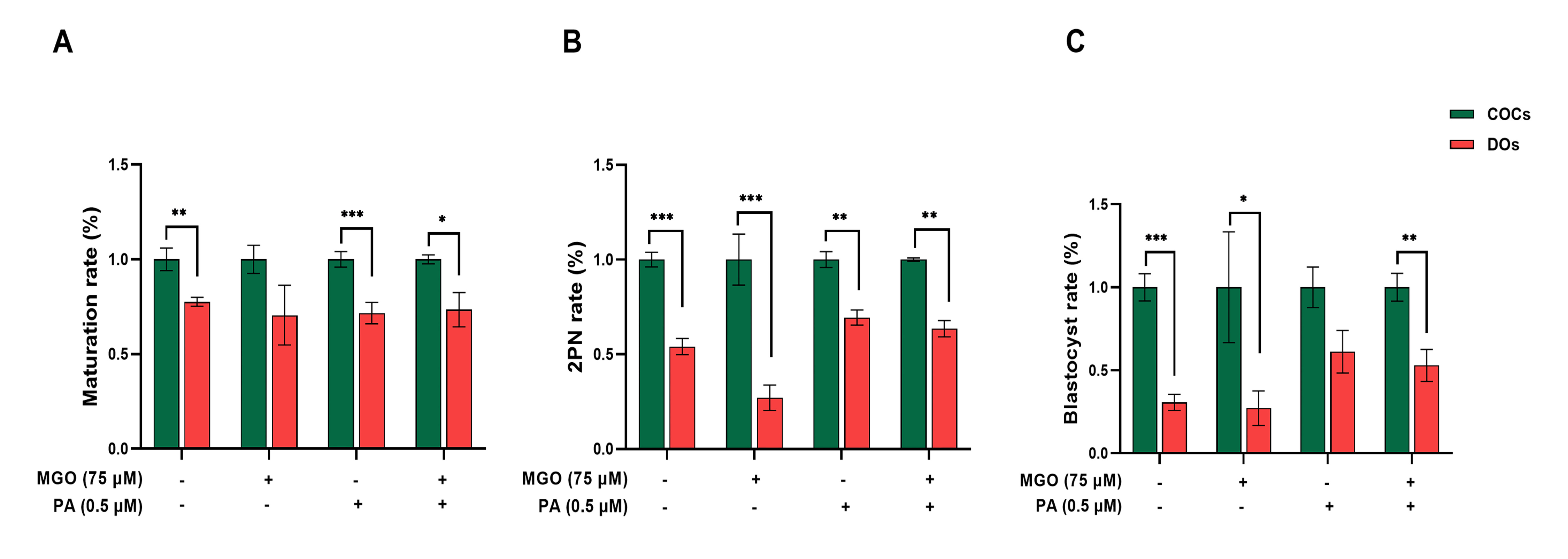

Supplement: S3 Fig — A) maturation rate, B) pronucleus formation rate and C) blastocyst rate. Data are presented as means ± SEM. Asterisks demonstrate significant differences between groups, Statistical differences between groups were assessed using independent sample t-test. * P < 0.05; ** P < 0.01 and *** P < 0.001. (TIF) [file pone.0314602.s004.tif]
